# Distinct Effects of Alcohol Consumption and Smoking on Genetic Alterations in Head and Neck Carcinoma

**DOI:** 10.1371/journal.pone.0080828

**Published:** 2013-11-20

**Authors:** Mitsuyoshi Urashima, Takanori Hama, Toshihito Suda, Yutaka Suzuki, Masahiro Ikegami, Chikako Sakanashi, Taisuke Akutsu, Suguru Amagaya, Kazuhumi Horiuchi, Yu Imai, Hidetoshi Mezawa, Miki Noya, Akio Nakashima, Aki Mafune, Takakuni Kato, Hiromi Kojima

**Affiliations:** 1 Division of Molecular Epidemiology, Jikei University School of Medicine, Tokyo, Japan; 2 Department of Oto-Rhino-Laryngology, Jikei University School of Medicine, Tokyo, Japan; 3 Department of Pathology, Jikei University School of Medicine, Tokyo, Japan; 4 Department of Surgery, International University of Health and Welfare, Tokyo, Japan; Virginia Commonwealth University, United States of America

## Abstract

**Background:**

Tobacco and alcohol consumption are risk factors for head and neck squamous cell carcinoma (HNSCC). Recently, whole-exome sequencing clarified that smoking increased TP53 and other mutations in HNSCC; however, the effects of alcohol consumption on these genetic alterations remain unknown. We explored the association between alcohol consumption and somatic copy-number alterations (SCNAs) across the whole genome in human papillomavirus (HPV)-negative HNSCCs, and compared with the effects of smoking on genetic alterations.

**Methods:**

SCNA and TP53 mutations in tumor samples were examined by high-resolution comparative genomic hybridization microarray 180K and by direct sequencing, respectively, and statistically analyzed for associations with alcohol consumption and smoking during the 20 years preceding diagnosis of HNSCC. Probes with a corrected p-value (=q-value) less than 0.05 and fold change greater than 1.2 or less than -1.2 were considered statistically significant.

**Results:**

A total of 248 patients with HNSCC were enrolled. In the HPV-negative patients (n=221), heavy alcohol consumption was significantly associated with SCNAs of oncogenes/oncosuppressors that were previously reported to occur frequently in HNSCCs: CDKN2A (q=0.005), FHIT (q=0.005), 11q13 region including CCND1, FADD and CTTN (q=0.005), ERBB2 (HER2) (q=0.009), 3q25-qter including CCNL1, TP63, DCUN1D1 and PIK3CA (q=0.014), and CSMD1 (q=0.019). But, TP53 mutations were not affected. In contrast, smoking was associated with increased risk of TP53 mutations, but did not induce any significant SCNAs of oncogenes/oncosuppressors.

**Conclusion:**

These results suggest that both alcohol consumption and smoking had distinct effects on genetic alterations in HNSCCs. Heavy alcohol consumption may trigger previously known and unknown SCNAs, but may not induce TP53 mutation. In contrast, smoking may induce TP53 mutation, but may not trigger any SCNAs.

## Introduction

Head and neck squamous cell carcinoma (HNSCC) arises from mucosa lining the paranasal sinuses, nasal cavities, oral cavity, nasopharynx, oropharynx, hypopharynx, and larynx. These anatomical sites can be directly exposed to extremely high levels of carcinogens via tobacco smoking or alcohol use. Indeed, at least 75% of HNSCC from the oral cavity, pharynx and larynx are attributed to smoking and/or alcohol use [1,2]. Human papillomavirus (HPV) is etiologically associated with some HNSCCs, mostly in oropharyngeal cancer [3], and HPV-positive HNSCCs are considered to have a distinct pathogenesis from HPV-negative HNSCCs [4].

Recently, whole-exome sequencing clarified that smoking increased TP53 and other mutations in HNSCC [5,6]. In contrast to smoking, the mechanisms by which alcohol consumption exerts its tumorigenic effect have not been fully elucidated [7]. Significant mutations in HNSCC associated with alcohol consumption have not been found, even by means of whole-exome sequencing [6]. In addition to single-base substitutions, somatic copy-number alterations (SCNAs) represent alternative mechanisms for cancer development. Analysis of 3,131 cancer specimens consisting of 26 histological types by application of high-resolution copy-number array resulted in the identification of both known and unknown cancer-related SCNAs without hypotheses [8]. In the present study, we explored the effects of alcohol consumption on SCNAs in HNSCC by using high-resolution comparative genomic hybridization (CGH) microarray. We then compared the effects of alcohol and tobacco use on SCNAs and TP53 mutations in HNSCC.

## Patients and Methods

### Study design

We conducted a prospective cohort study at Jikei University Hospital from March 2006 to November 2012. The study-protocol was reviewed and approved by the Ethics Committee for Biomedical Research of the Jikei Institutional Review Board. The entire process of study design, data monitoring, and analyses was performed at the Division of Molecular Epidemiology. Eligible participants were at least 20 years old, had newly diagnosed or recurrent HNSCC, and had surgical resection with curative intent before chemoradiotherapy. All of the consecutive patients provided written informed consent.

Clinical information was obtained from clinical and surgical charts. Tumor node metastasis (TNM) classification and cancer stages were determined according to the 6th Union for International Cancer Control TNM classification and stage groupings.

### Alcohol consumption and smoking

Patients were divided into the following three categories based upon average daily alcohol consumption during the 20 years preceding diagnosis of HNSCC: 1) Non-drinkers, defined as non-drinkers or light drinkers who consumed less than one drink per day; 2) Moderate drinkers, defined as drinkers who consumed at least one but less than two drinks per day, and 3) Heavy drinkers, defined as drinkers who consumed two or more drinks per day. One drink was defined as containing approximately 10 g of alcohol, which is equal to 30 ml of hard liquor, 100 ml of wine containing 12% alcohol, or 360 ml of beer.

Patients were divided into the following three groups based on smoking history prior to diagnosis: 1) Non-smokers, defined as patients who had never used tobacco or had stopped using tobacco for more than 20 years; 2) Moderate smokers, defined as current or past smokers who smoked less than 20 pack-years within the last 20 years; and 3) Heavy smokers, defined as current or past smokers who smoked at least 20 pack-years within the last 20 years.

### Tissue samples

During surgery, tumor and margin samples from the primary site, but not metastatic sites, were collected. These samples were rapidly frozen and stored at –80°C after excision. The cancer tissue was divided into the following two specimens: one for pathological confirmation, where the tumor sample was composed of more than 70% cancer cells; and the other for DNA extraction. Peripheral blood samples were also collected to confirm whether the genetic alterations in the tumors were due to genomic polymorphisms or somatic mutations.

### Human papillomavirus detection

The detection of nine HPV high-risk types (16/18/31/33/35/52b/58) was performed by multiplex polymerase chain reaction (PCR) by TaKaRa Human Papillomavirus Typing Set #6603 following the manufacturer’s protocol (Takara Bio Inc. Otsu, Shiga, Japan). 

### Array-based comparative genome hybridization (CGH)

For array-based CGH, 0.5 μg genomic DNA was labeled with the Agilent Enzymatic labeling kit. Then, labeled DNA was hybridized to the Agilent-022060 SurePrint G3 Human CGH Microarray 4×180K (Agilent Technologies Inc. Santa Clara, CA) and scanned by using the Agilent Microarray Scanner with FEATURE EXTRACTION v. 10.7.3.1 (Agilent Technologies Inc.). These data were subsequently imported into GeneSpringGX v.11.5.1 (Agilent Technologies Inc.) for analysis.

Normalization was performed by using the Log_2_ signal ratio (sample DNA/control DNA) for individual 180K probes distributed throughout the whole genome. Control DNA was obtained from a single Japanese person, who is one of authors (MU).

When GeneSpringGX v.11.5.1 was used to compare data between two groups (e.g., non-drinkers vs. heavy drinkers), an unpaired t test was used and subsequently corrected by the Benjamini-Hochberg false-discovery rate. Probes with a corrected p-value (=q-value) less than 0.05 and fold change greater than 1.2 or less than -1.2 were considered statistically significant. Chromosomes X and Y were excluded from the analyses, because smoking and alcohol habits are seriously deviated to men. The study data have been deposited in NCBIs Gene Expression Omnibus (http://www.ncbi.nlm.nhi.gov/geo/, series accession number GSE47443).

### TP53 mutation

Exons 2 to 11 of the TP53 gene were amplified by PCR with purchased primers following the manufacturer’s protocol (Nippon Gene Co. Ltd., Chiyoda-ku, Tokyo, Japan), cloned, and then sequenced with the ABI PRISM 3700 Genetic Analyzer (Applied Biosystems, Foster City, CA). Disruptive TP53 mutations are non-conservative mutations located inside the key DNA-binding domain (L2-L3 region) or stop codons in any region [9]. Missense changes V31I, P36P, P47S, P72R, R72R, R158R, R213R, V217M, P222P, T312S, and G360A were reported as single nucleotide polymorphisms [10] and thus excluded from total p53 mutations.

### p16 immunohistochemistry

Formalin-fixed, paraffin-embedded tumor specimens were evaluated for p16 expression with a rabbit monoclonal antibody to p16 (Anti-CDKN2A/p16INK4a antibody [EPR1473]): Abcam plc, Science Park, Cambridge, England). Positive p16 expression in immunohistochemistry (IHC) was defined as strong and diffuse nuclear and cytoplasmic staining in at least 70% of tumor cells.

### Statistical analysis

The ratio of patients who had SCNA and TP53 mutations was compared between drinking and smoking groups by using the risk ratio (RR) and risk difference (RD) with 95% confidence interval (CI). All statistical analyses were performed with STATA 12.1 (STATA Corp., College Station, TX). P < 0.05 was considered statistically significant.

## Results

### Patient characteristics by HPV-positivity and primary sites

A total of 262 patients were registered after giving informed consent. Fourteen patients were withdrawn because the pathological diagnosis was not HNSCC (N=12) or the primary tumor site was unknown (N=2). Clinical data from the remaining 248 patients were used. CGH array could not be performed for 15 patients due to insufficient cellular volume and for 8 patients due to insufficient DNA quality. Stages of these 23 patients tended to be earlier than the other patients (P=0.001).

A total of 248 patients were first stratified into HPV-positive and HPV-negative groups. HPV-negative patients were further stratified by primary tumor sites. Patient characteristics were compared among these subgroups (Table 1). Patients with oral and laryngeal cancer had relatively earlier stages and well differentiated pathology as compared to other patients. Second primary cancers were more common in patients with oropharyngeal and hypopharyngeal cancer than in other patients. The prevalence of smokers and alcohol drinkers was 71% and 62%, respectively. Non-smokers and non-drinkers were significantly more common in HPV-positive, sinonasal, and oral cancer patients than in other patients. TP53 mutations were detected in 63% of total tumor samples; this percentage was lower in HPV-positive patients (31%) and higher in HPV-negative patients with oropharyngeal (77%) and hypopharyngeal (77%) cancer.

**Table 1:** Patient characteristics stratified by HPV positivity and primary tumor sites^***a***^.

	Total	HPV-positive patients**^*†*^**			HPV-negative patients**^*b*^**			p-value
	n=248	n=27			n=221			
			Sino-nasal	Oropharynx	Hypopharynx	Larynx	Oral cavity	
			n=23	n=41	n=65	n=33	n=59	
Age, years – yr. mean ± s.d.	63.2±11.0	58.6±10.1	61.7±12.8	65.2±11.3	66.9±6.7	66.3±10.6	58.7±12.5	0.0001**^*c*^**
Women – no. (%)	51 (21)	6 (22)	7 (30)	6 (15)	9 (14)	3 (9)	20 (34)	0.022**^*d*^**
Stage – no. (%)								< 0.001**^*d*^**
I	17 (7)	0 (0)	0 (0)	1 (2)	3 (5)	5 (15)	8 (14)	
II	49 (20)	4 (15)	2 (9)	14 (34)	8 (12)	5 (15)	16 (28)	
III	52 (21)	5 (19)	10 (43)	9 (22)	8 (12)	7 (21)	13 (23)	
IV	128 (52)	18 (67)	11 (48)	17 (41)	46 (71)	16 (48)	20 (35)	
Cell differentiation – no. (%)								
Well differentiated	74 (31)	5 (19)	7 (32)	8 (20)	11 (18)	15 (48)	28 (50)	0.001**^*d*^**
Moderately differentiated	118 (49)	16 (59)	8 (36)	25 (61)	34 (55)	11 (35)	24 (43)	
Poorly differentiated	47 (20)	6 (22)	7 (32)	8 (20)	17 (27)	5 (16)	4 (7)	
Second primary cancers	48 (19)	3 (11)	2 (9)	16 (39)	19 (29)	2 (6)	6 (10)	< 0.001**^*d*^**
Tobacco smoke								
Pack-year – median (IQR**^*e*^**)	25 (0 - 40)	10 (0 - 30)	19 (0 - 40)	30 (20 - 50)	32 (5 - 49)	40 (14 - 40)	15 (0 - 40)	0.0006**^*f*^**
Smoking status – no. (%)								< 0.001**^*d*^**
Non-smoker	71 (29)	11 (41)	9 (41)	5 (12)	16 (25)	4 (12)	26 (44)	
Moderate smoker	28 (11)	7 (26)	2 (9)	5 (12)	1 (2)	6 (18)	7 (12)	
Heavy smoker	147 (60)	9 (33)	11 (50)	31 (76)	47 (73)	23 (70)	26 (44)	
Drinking status – no. (%)								0.015**^*d*^**
Non-drinker	93 (38)	14 (52)	10 (45)	14 (34)	17 (26)	11 (33)	27 (46)	
Moderate drinker	77 (31)	2 (7)	9 (41)	13 (32)	19 (29)	14 (42)	20 (34)	
Heavy drinker	77 (31)	11 (41)	3 (14)	14 (34)	29 (45)	8 (24)	12 (20)	
Smoking/drinking – no. (%)								0.001**^*d*^**
Non-smoker non-drinker	53 (22)	8 (30)	8 (38)	4 (10)	10 (16)	3 (9)	20 (34)	
Smoker but non-drinker	39 (16)	6 (22)	2 (10)	10 (24)	6 (9)	8 (24)	7 (12)	
Drinker but non-smoker	18 (7)	3 (11)	1 (5)	1 (2)	6 (9)	1 (3)	6 (10)	
Both smoker and drinker	135 (55)	10 (37)	10 (48)	26 (63)	42 (66)	21 (64)	26 (44)	
TP53 mutations – no. (%)								0.001**^*d*^**
Wild-type TP53	87 (37)	18 (69)	9 (43)	9 (23)	15 (23)	12 (41)	24 (44)	
Non-disruptive TP53	94 (40)	5 (19)	6 (29)	15 (38)	36 (56)	13 (45)	19 (35	
Disruptive TP53	53 (23)	3 (12)	6 (29)	15 (38)	13 (20)	4 (14)	12 (21)	

^a^ Because of rounding, the sum of totals is not always 100%. Patients with non-squamous cell cancer or unknown primary sites were excluded. Smoking and drinking status were unknown in 3 patients.

^b^ A total of 248 patients were stratified into HPV-positive patients and HPV-negative patients. Then, HPV-negative patients were divided by the primary sites.

^c^ P-value was calculated by ANOVA.

^d^ P-value was calculated by χ^2^ test.

^e^ Interquartile range

^f^ Kruskal-Wallis equality-of-populations rank test

### Genome-wide screening for significant SCNAs associated with alcohol consumption and smoking

**Figure 1:**
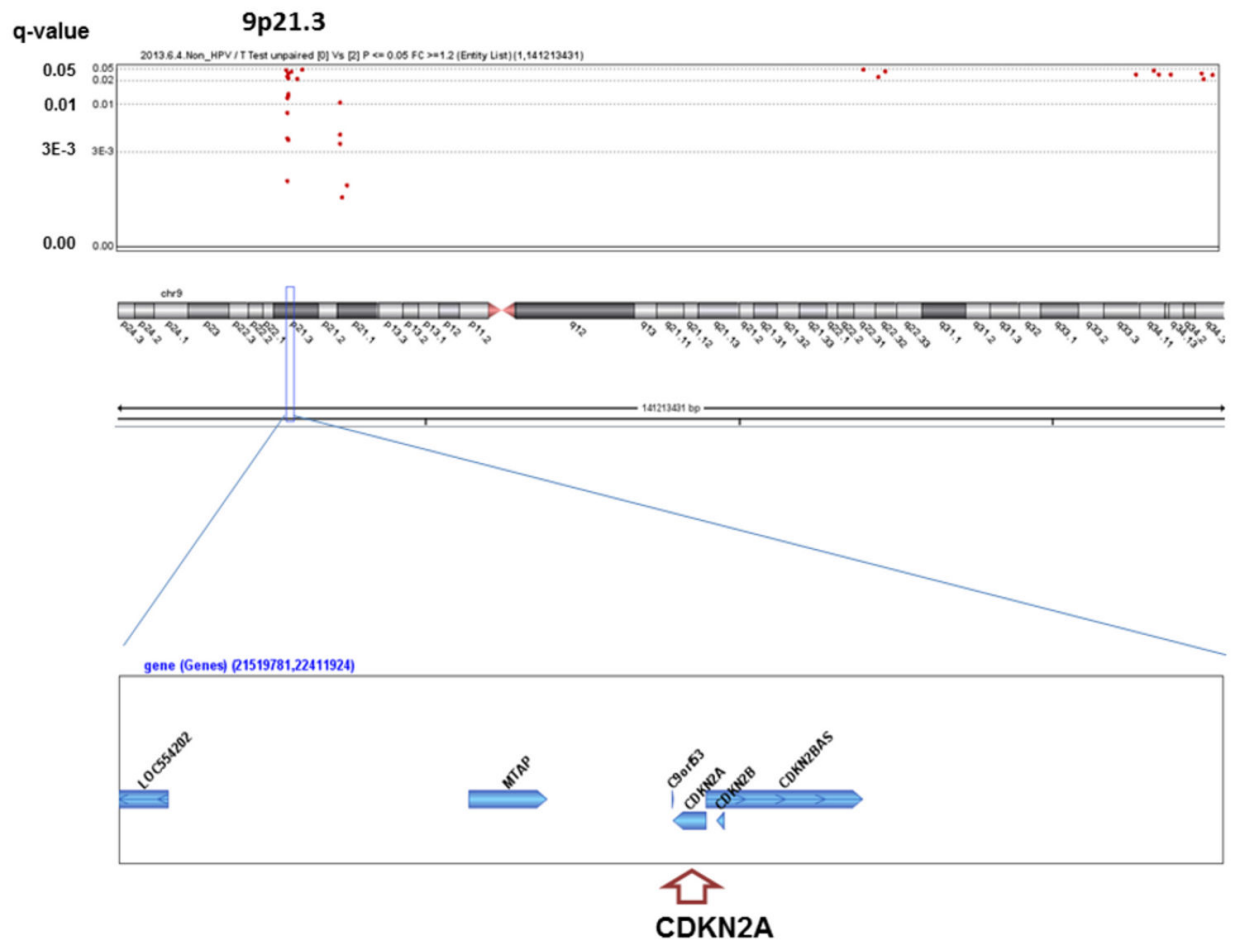
Significant SCNAs at 9p21.3 associated with heavy alcohol consumption.

**Figure 2:**
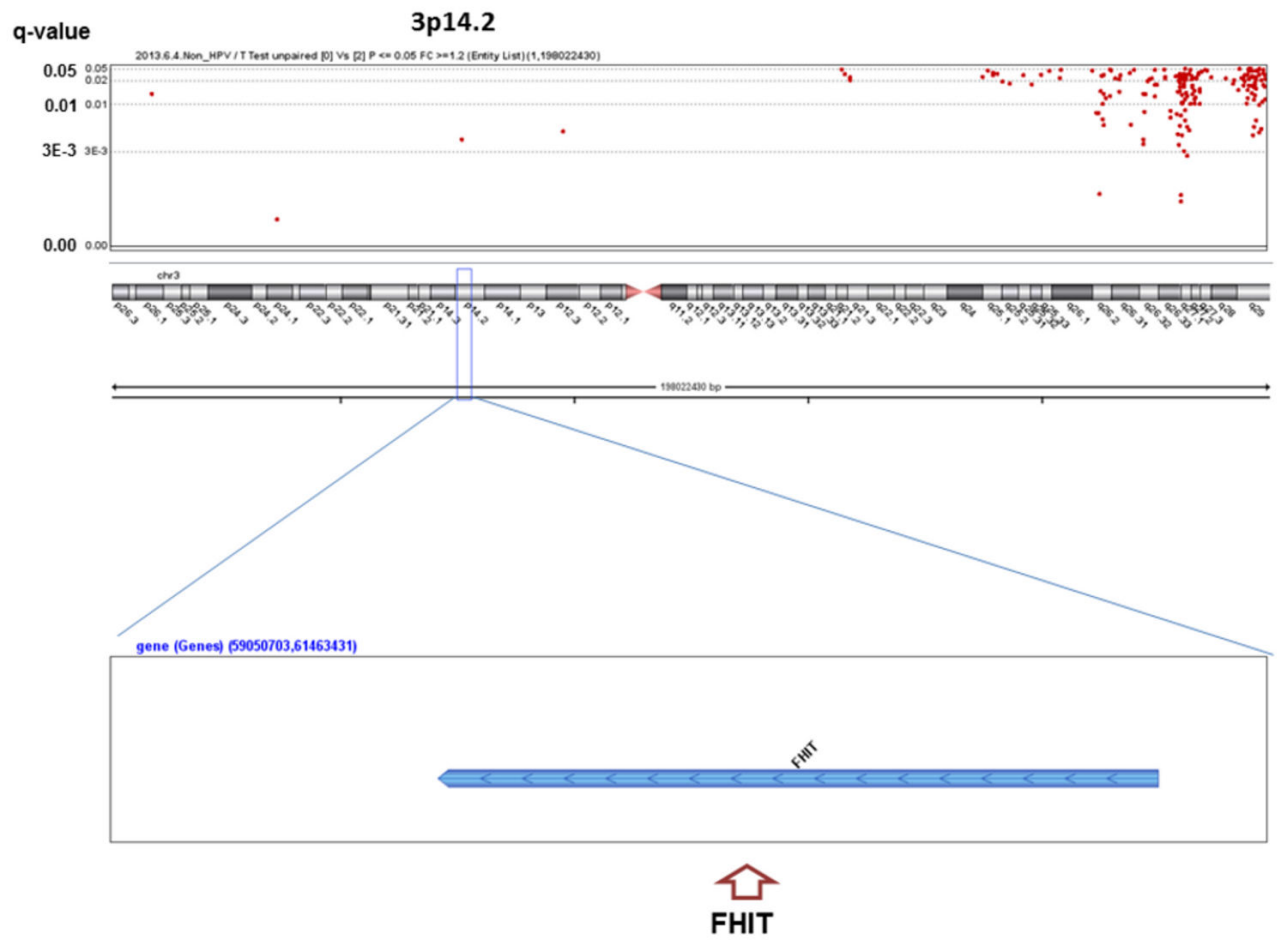
Significant SCNAs at 3p14.2 associated with heavy alcohol consumption.

**Figure 3:**
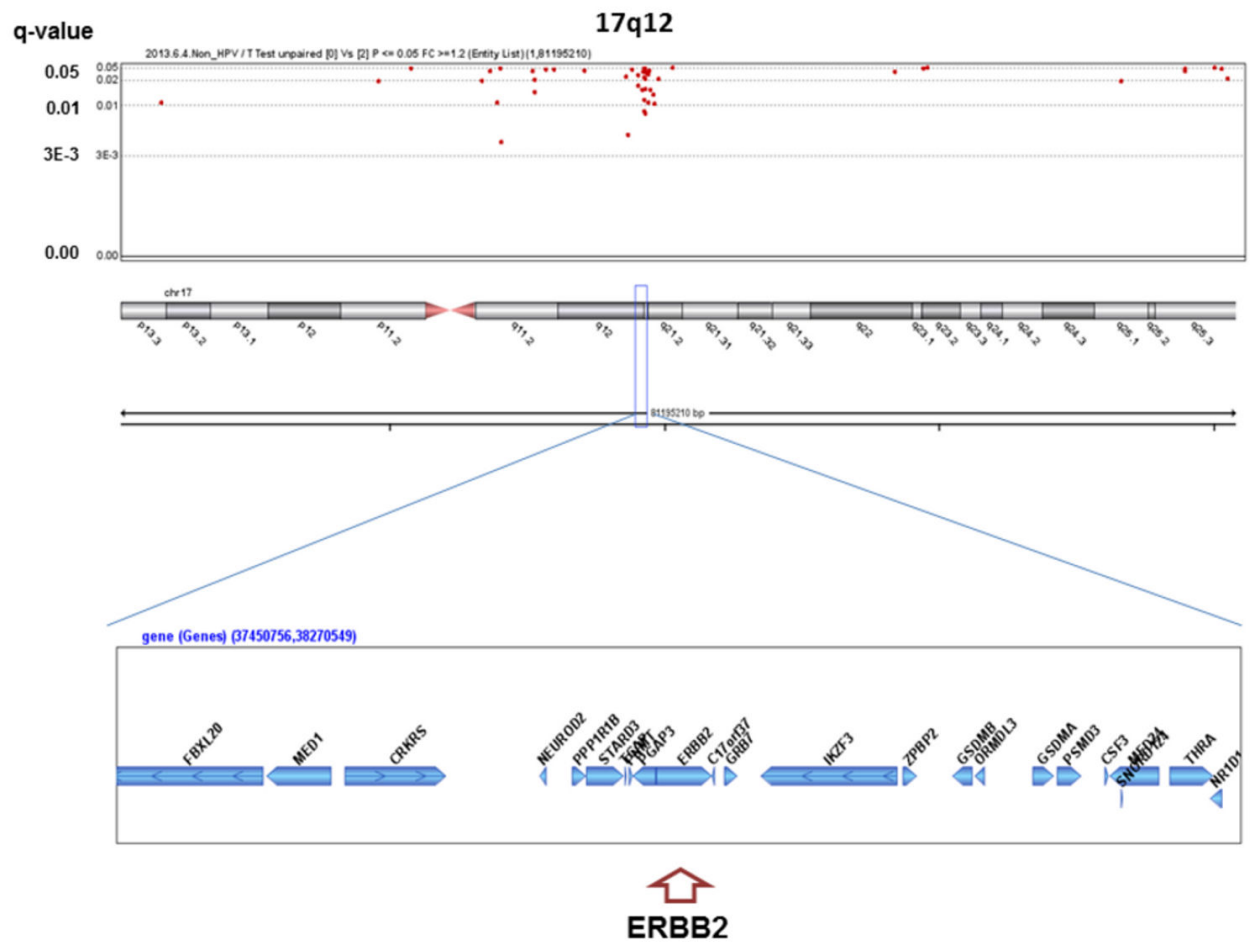
Significant SCNAs at 17q12 associated with heavy alcohol consumption.

**Figure 4:**
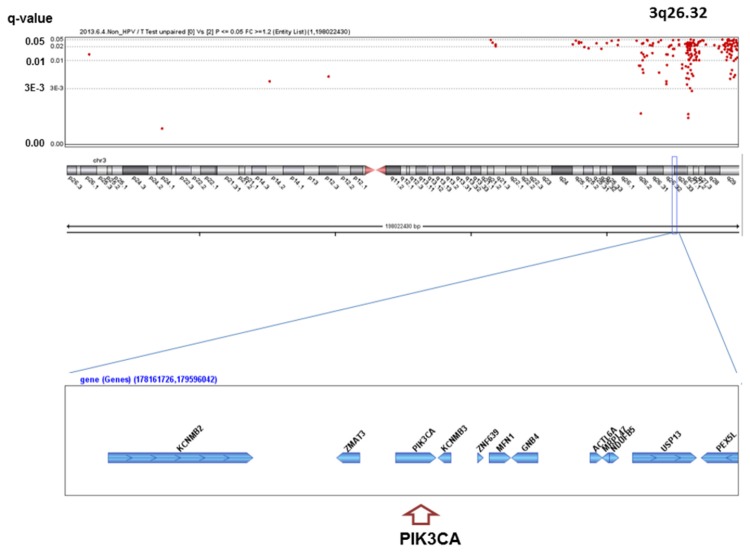
Significant SCNAs at 3q26.32 associated with heavy alcohol consumption.

**Figure 5:**
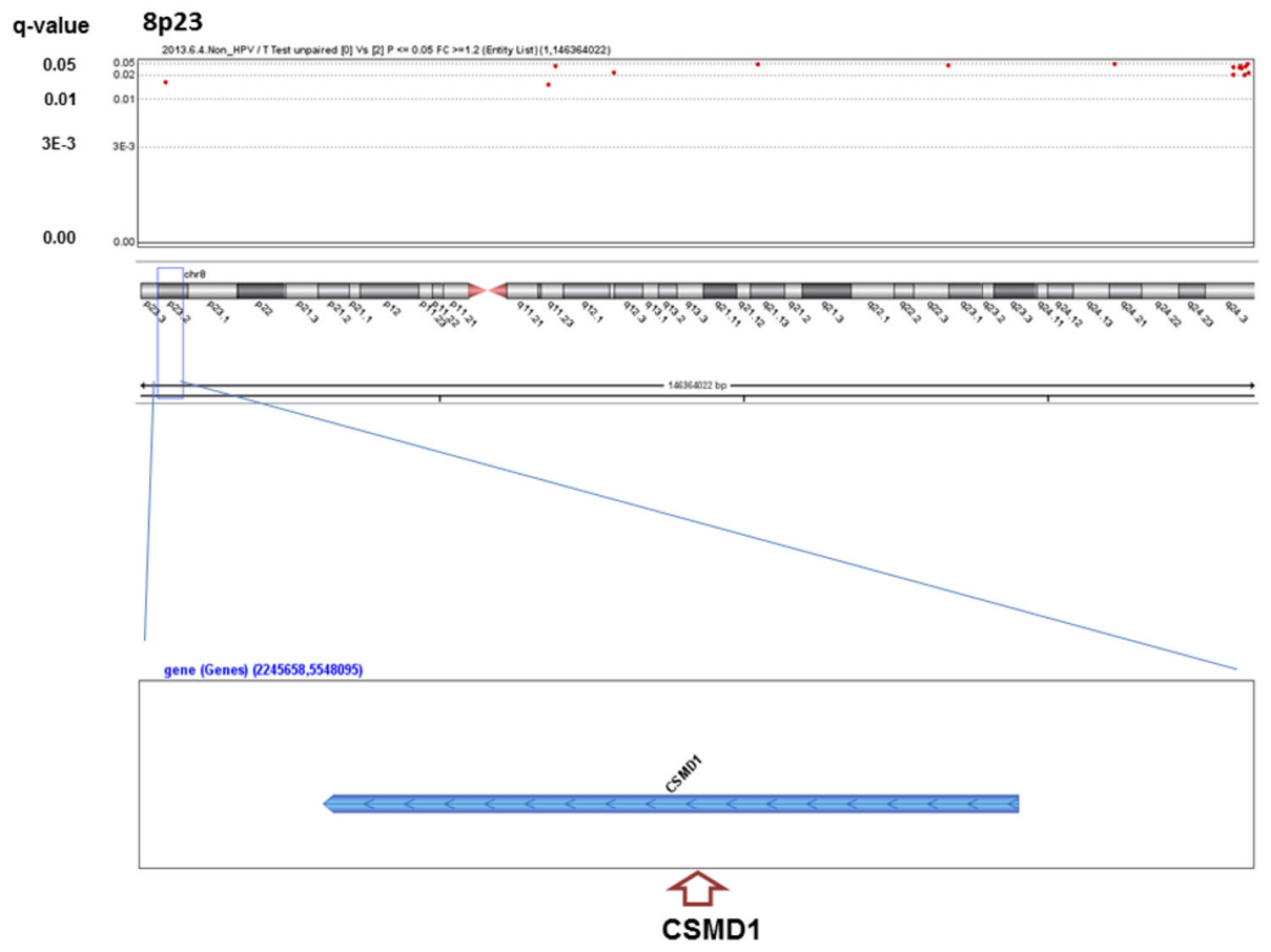
Significant SCNAs at 8p23 associated with heavy alcohol consumption.

**Figure 6:**
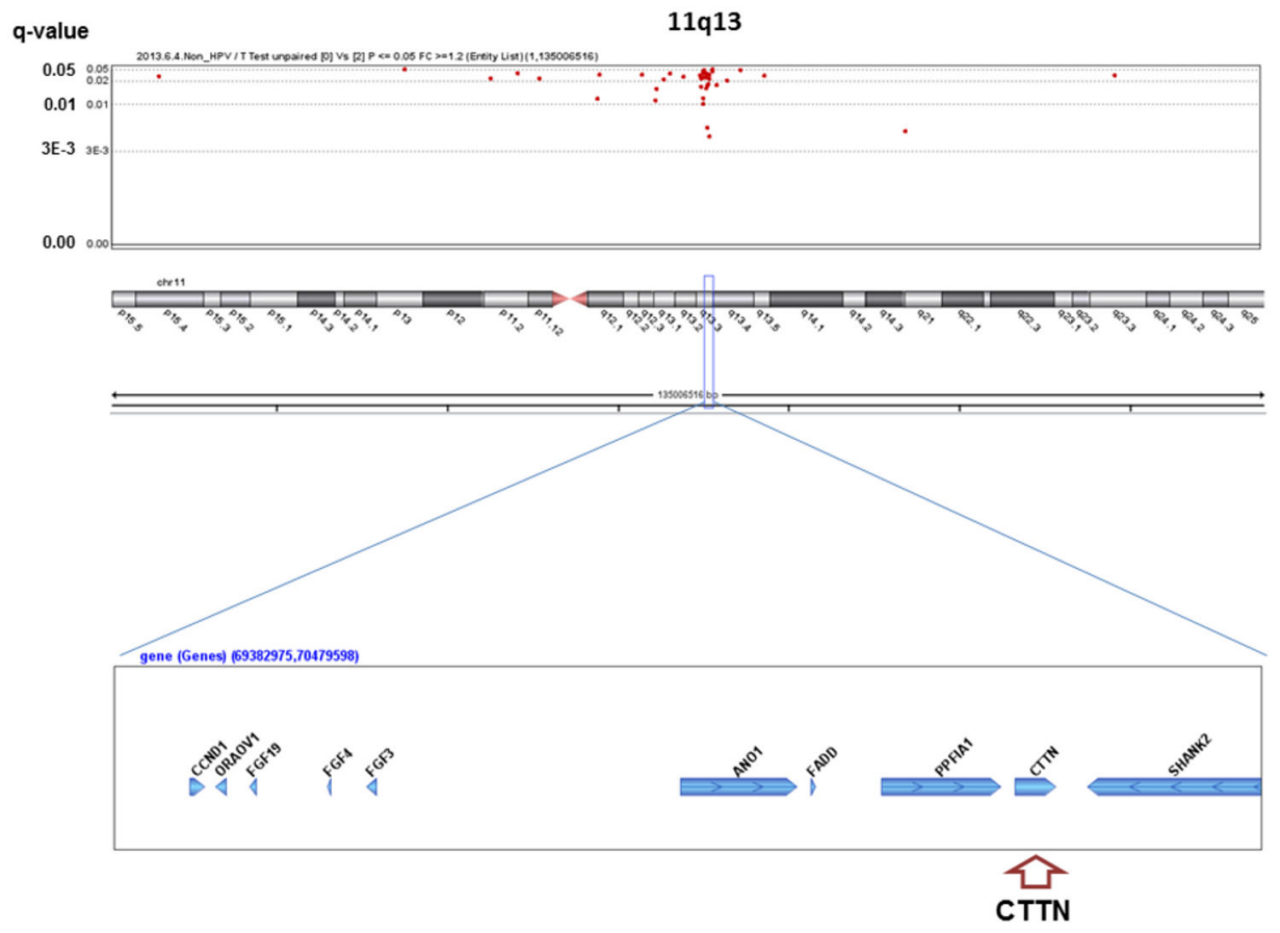
Significant SCNAs at 11q13 associated with heavy alcohol consumption. Significant q-values (<0.05) (y-axis) are plotted across the chromosome (x-axis). An oncogene or oncosuppressor gene associated with development of HNSCCs in previous studies is indicated in parentheses in each peak region. X and Y chromosomes were excluded for analysis, because alcohol drinkers and smokers were predominantly men.

We analyzed the q-values of SCNAs associated with alcohol-drinking status. No significant SCNAs were found in the HPV-positive patients, whereas 363 significant SCNAs were detected between heavy drinkers and non-drinkers in the HPV-negative patients (Table S1), including well-known oncogenes or oncosuppressors in HNSCC (Figure 1~6). The most significant SCNAs were deletions at the 9p21.3 region, including CDKN2A (q=0.005) and CDKN2B (q=0.015) oncosuppressor genes, which produce p16 and p15 protein, respectively (Figure 1). Deletion of CDKN2A was confirmed by p16 protein immunohistochemistry. The second was down-regulation of 3p14.2 including FHIT (q=0.005) (Figure 2), and the third was amplification at 17q12 including ERBB2 (or HER2) (q=0.009) (Figure 3). The fourth was a relatively broad amplified region around 3q25-qter including PIK3CA (q=0.014) (Figure 4), CCNL1, TP63, and DCUN1D1. The fifth was down-regulation of 8p23 including CSMD1 (q=0.019) (Figure 5). The sixth was amplification at 11q13.3 including CCND1; ORAOV1; FGF19 (q=0.010); FGF4; FGF3; ANO1 (q=0.007); FADD; PPTIA1 (q=0.022); CTTN (q=0.005); and SHANK2 (q=0.001) (Figure 6). However, no such significant SCNAs were identified between moderate drinkers and non-drinkers.

We analyzed q-values of SCNAs based on smoking status (non-smokers vs. smokers; non-smokers vs. moderate smokers vs. heavy smokers). However, no significant SCNAs of oncogenes or oncosuppressors were detected, similar to results observed for alcohol consumption. When patients who both smoked and drank alcohol were compared to patients who did not, no significant SCNAs were found.

### Effects of alcohol consumption on SCNAs and TP53 mutations in HPV-negative patients

By evaluating individual CGH data of 199 HPV-negative patients, the SCNAs were further confirmed to keep associations with alcohol consumption status (Table 2). CDKN2A gene alterations (P<0.0001) and homogeneous deletion (P<0.0001) occurred significantly more frequently in heavy drinkers than in non-drinkers and moderate drinkers. ERBB2 gene amplification occurred in 0% of non-drinkers, 6% of moderate drinkers and 13% of heavy drinkers. Heavy drinkers had significantly more ERBB2 amplification than non-drinkers and moderate drinkers (P=0.005). FHIT and CSMD1 gene deletion, as well as PIK3CA gene amplification, were more frequent in heavy drinkers than in other patients. The amplified CCND1 gene was slightly more frequent in heavy drinkers than the other groups, but showed strong association with CDKN2A gene alteration (RR, 4.64; 95%CI, 2.39 to 7.19, P<0.0001). Furthermore, significant differences were observed between drinkers and non-drinkers in CDKN2A, ERBB2, FHIT and CSM1 genes. Frequencies of TP53 mutations were statistically the same among non-drinkers, moderate drinkers, and heavy drinkers.

**Table 2:** Effects of alcohol drinking on SCNAs, TP53 mutations in HPV-negative patients.

		Alcohol drinkers		Heavy drinkers vs. moderate ~ non-drinkers		Drinkers vs. non-drinkers	
	None	Moderate	Heavy	RR (95%CI)	p-value	RR (95%CI)	p-value
	N=72	N=66	N=61	RD (95%CI)		RD (95%CI)	
CDKN2A**^*a*^** gene deletions: 1 copy or 0 copy – no. (%) total: 49 (25%)	12 (17)	10 (15)	27 (44)	2.78 (1.73 to 4.47)	< 0.0001	1.75 (0.98 to 3.31)	0.050
				0.28 (0.14 to 0.42)		0.12 (0.01 to 0.24)	
Homogeneous deletion of CDKN2A gene:0 copy – no. (%) total: 12 (6%)	1 (1)	1 (2)	10 (16)	11.31 (2.55 to 50.1)	< 0.0001	6.24 (0.82 to 47.3)	0.038
				0.15 (0.05 to 0.24)		0.08 (0.07 to 0.17)	
ERBB2**^*b*^** amplification: 4 copies ≤ –no. (%) total: 12 (6%)	0 (0)	4 (6)	8 (13)	4.52 (1.42 to 14.5)	0.005	-	0.007
				0.10 (0.01 to 0.19)		0.09 (0.04 to 0.15)	
FHIT**^*c*^** gene deletions: 1 copy or 0 copy – no. (%) total: 42 (21%)	8 (11)	14 (21)	20 (33)	2.06 (1.22 to 3.48)	0.007	2.41 (1.18 to 4.92)	0.009
				0.17 (0.04 to 0.30)		0.16 (0.05 to 0.26)	
CSMD1**^*d*^** gene deletions: 1 copy or 0 copy – no. (%) total: 38 (19%)	7 (10)	9 (14)	22 (36)	3.11 (1.76 to 5.50)	0.0001	2.51 (1.17 to 5.40)	0.011
				0.24 (0.11 to 0.38)		0.15 (0.05 to 0.25)	
PIK3CA**^*e*^** amplification: 3 copies ≤ – no. (%) total: 92 (47%)	30 (42)	25 (39)	37 (64)	1.58 (1.19 to 2.09)	0.003	1.22 (0.88 to 1.69)	0.22
				0.23 (0.08 to 0.38)		0.09 (-0.05 to 0.24)	
CCND1**^*f*^** amplification: 4 copies ≤ – no. (%) total: 39 (20%)	10 (14)	11 (17)	18 (30)	1.94 (1.12 to 3.37)	0.019	1.64 (0.85 to 3.17)	0.13
				0.14 (0.01 to 0.27)		0.09 (-0.02 to 0.20)	
TP53 mutation – no. (%) Total: 139 (67%)	45 (60)	54 (75)	40 (66)	0.97 (0.79 to 1.21)	0.80	1.18 (0.95 to 1.46)	0.12
				-0.02 (-0.16 to 0.12)		0.11 (-0.03 to 0.24)	

^a^ CDKN2A: cyclin-dependent kinase inhibitor 2A

^b^ ERBB2: v-ERB-B2 avian erythroblastic leukemia viral oncogene homologue2

^c^ FHIT: fragile histidine triad gene

^d^ CSMD1: cub and sushi multiple domains1

^e^ PIK3CA: phosphatidylinositol3-kinase catalytic alpha (q=0.014)

^f^ CCND1: cyclin D1

### Effects of smoking on TP53 mutations

In the overall study population, TP53 mutations were detected slightly but significantly more in smokers (66%) than in non-smokers (52%) (P=0.045). When we used a cutoff point of 10 pack-years, the risk of TP53 mutation was still significant (P=0.030). However, when the cutoff point was increased to 20 pack-years and more, the significance was lost.

## Discussion

We found that alcohol consumption and smoking had distinct effects on genetic alterations in HNSCCs. Heavy alcohol consumption triggered a variety of SCNAs, including oncogenes and oncosuppressor genes associated with the development of HNSCCs in HPV-negative patients, but did not affect TP53 mutations, which is a novel finding. In contrast, smoking raised the risk of TP53 mutations, which is consistent with Brennan’s report [11], but did not affect SCNAs, which also represents a novel finding. Smeets et al. reported that oropharyngeal cancer patients with hardly any chromosomal aberrations had significant associations with non-alcohol drinkers [12], which is consistent with our results.

Of interest, heavy drinking but not moderate drinking had significant effects on the induction of SCNAs. On the other hand, the TP53 mutation risk was increased in smokers and was not always significant in heavy smokers with more than 20 pack-years. According to Hashibe’s report [1], alcohol consumption was associated with an increased risk of HNSCCs only when consumed at a high frequency, suggesting a threshold model, whereas even a small amount of smoking raised the risk, suggesting a stochastic model of carcinogenesis.

Some of the SCNAs identified by CGH array as significantly associated with heavy alcohol consumption were matched with regions or genes frequently altered in HNSCC: 3p14 of FHIT, 8p23 of CSMD1, 9p21 of CDKN2A, 3q25-26 including CCNL1, PIK3CA, TP63 and DCUN1D1, and 11q13 including CCND1, CTTN and FADD [13-15]. Of these typical SCNAs in HNSCC, only CDKN2A was previously reported as a significant gene associated with heavy alcohol consumption, but not with smoking by Kraunz et al. [16]. However, associations between alcohol consumption and other SCNAs of the ERBB2, FHIT and CSMD1 genes, as well as the 11q13 and 3q25-26 regions, represent original findings.

Among the HPV-negative patients, ERBB2 gene amplification occurred only in moderate and heavy alcohol drinkers. In a murine model, ERBB2 expression was increased in alcohol-exposed mucosa, dysplasia, and invasive oral carcinomas [17], which may support our results. Concerning esophageal [18] and breast cancer [19], in both of which alcohol consumption is a risk factor [7] and ERBB2 gene amplification was reported. 

 In summary, both alcohol consumption and smoking had distinct effects on genetic alterations in HNSCCs. Heavy alcohol consumption may trigger previously known and unknown SCNAs, but may not induce TP53 mutation. In contrast, smoking may induce TP53 mutation, but may not trigger any SCNAs. 

## Supporting Information

Table S1 **Significant SCNAs detected between heavy drinkers and non-drinkers in the HPV-negative patients.**
(XLSX)
